# Beyond Diagnosis: Characterising Symptom Profiles of Dementia in a Resource-Limited South African Clinical Setting

**DOI:** 10.3390/healthcare14152365

**Published:** 2026-08-03

**Authors:** Mphumzi Andreas Dlamini, Laston Gonah, Sibusiso Cyprian Nomatshila

**Affiliations:** 1 Department of Psychology, Faculty of Law, Humanities and Social Sciences, iYunivesithi Walter Sisulu, Mthatha 5117, South Africa; 2 School of Public Health, Faculty of Medicine and Health Sciences, iYunivesithi Walter Sisulu, Sisson Street, Mthatha 5117, South Africa; 3 Society and Health Research Institute, Faculty of Medicine and Health Sciences, iYunivesithi Walter Sisulu, Sisson Street, Mthatha 5117, South Africa

**Keywords:** dementia, prevalence, memory loss, behavioural symptoms, comorbidity, record review, associated factors

## Abstract

**Background**: The demographic and clinical heterogeneity of dementia is well recognised; however, the consistency of core symptom profiles across age and gender in clinical populations remains underexplored. This study aimed to determine the burden and distribution of dementia-related symptoms in selected rural health facilities in South Africa. **Methods**: A retrospective cross-sectional study was conducted among 260 patients with a diagnosed dementia condition. Data on demographics and key symptoms (memory loss, short-term memory loss, behavioural changes, delusions and communication difficulties), as well as chronic disease comorbidities, were extracted from electronic health records. Associations were assessed using Chi-square tests. **Results**: The cohort was predominantly female (59.6%) and older (64.3% aged ≥71 years). Symptom prevalence was high: short-term memory loss and behavioural changes (96.9% each), general memory loss (91.2%), chronic disease comorbidity (75.8%), communication difficulties (67.3%), and delusions (57.7%). No statistically significant associations were found between any of the dementia symptoms and age or gender (*p* > 0.05), indicating a uniformly high symptom burden across demographic subgroups. **Conclusions**: Dementia-related symptoms and comorbidities were consistently high and severe in this clinical population, irrespective of age or gender. These findings underscore the need for integrated, person-centred care models addressing both cognitive and physical health in resource-limited settings.

## 1. Introduction

Dementia is a major global public health challenge, characterised by progressive cognitive decline that interferes with independent functioning and daily living activities [1]. With population ageing, its prevalence is projected to increase substantially, placing growing pressure on healthcare systems and caregivers [2]. Currently, an estimated 55 million people worldwide are affected, a number expected to triple by 2050 [3]. Dementia is a syndrome characterised by deterioration in cognitive domains beyond what would be expected from normal ageing, affecting memory, thinking, orientation, comprehension, calculation, learning capacity, language, and judgment [4].

The clinical presentation of dementia is heterogeneous, reflecting diverse underlying pathologies such as Alzheimer’s disease, vascular dementia, and frontotemporal disorders [5,6]. Common symptoms include memory loss, behavioural changes, communication difficulties, and neuropsychiatric disturbances. Previous studies suggest that symptom expression may vary by age and gender; however, evidence remains inconsistent, particularly in low- and middle-income settings [7,8]. This variability underscores the need to better understand whether symptom patterns differ meaningfully across demographic groups in real-world clinical populations.

Age is the strongest known risk factor for most forms of dementia, with genetic factors contributing to disease development. Early-onset dementia (<65 years) is often associated with mutations in APP, PSEN1, PSEN2 (Alzheimer’s disease), and MAPT and GRN (frontotemporal dementia), typically with an autosomal dominant pattern [9,10]. Late-onset dementia involves a more complex genetic architecture, with APOE ε4 as a well-established but non-deterministic risk factor [11]. Collectively, these determinants highlight the multifactorial and heterogeneous nature of dementia.

In addition to cognitive decline, multimorbidity is highly prevalent among individuals with dementia and complicates disease management [12]. Chronic conditions such as hypertension, diabetes, and cardiovascular disease may accelerate cognitive decline and increase care complexity [13]. The coexistence of dementia with other chronic conditions places substantial demands on healthcare systems, particularly in settings with limited resources.

Despite this, most evidence comes from high-income countries and often focuses on isolated symptom domains. Research examining overall symptom burden and demographic distribution in real-world, resource-limited clinical settings remains limited.

While core cognitive deficits define the syndrome, associated neuropsychiatric symptoms and functional impairments are often the primary determinants of caregiver burden, reduced quality of life, and institutionalisation [14,15]. Understanding the prevalence and distribution of this complex symptomatology across cognitive, behavioural, psychiatric, and somatic domains is therefore critical for effective health system planning, resource allocation, and the design of targeted interventions.

Most existing research on dementia has focused on specific symptom domains or narrow patient subgroups, predominantly in high-income countries [16]. Consequently, there remains a significant evidence gap regarding the full clinical spectrum of dementia in under-resourced settings. A holistic assessment of dementia-related symptoms and comorbidities in real-world clinical populations is notably lacking.

Therefore, this study aimed to address this gap by providing a comprehensive assessment of dementia symptom burden and its demographic distribution in a resource-limited clinical setting. Specifically, the study sought to (1) describe the prevalence of a broad range of dementia-related symptoms and comorbid conditions in a clinical population; and (2) examine the associations between these features and key demographic variables, namely age and gender. We hypothesised that while core cognitive symptoms would be highly prevalent, the distribution of neuropsychiatric symptoms and comorbidities would vary across demographic groups.

## 2. Materials and Methods

### 2.1. Study Design and Setting

A retrospective record review study was conducted to examine the clinical characteristics of patients diagnosed with dementia at OR Tambo health facilities between 2019 and 2024. This design enabled the assessment of symptom patterns and comorbidities using routinely collected data in a real-world, resource-limited healthcare setting.

### 2.2. Participants and Data Collection

The study included 260 patients with a confirmed diagnosis of dementia. Eligible records were identified based on documented clinical diagnoses made by healthcare professionals, and only records from 2019 to 2024 were included. This period was selected because it corresponded with the most complete and consistently documented patient records across the facilities, likely reflecting improvements in record-keeping practices, increased availability of trained personnel, and enhanced clinical documentation systems. Records outside this period were excluded due to greater levels of incomplete or inconsistent data, which could have compromised the reliability of the analysis.

Data on demographic characteristics, including age, gender, and year of diagnosis, as well as key dementia-related symptoms (memory loss, short-term memory loss, behavioural changes, delusions, and communication difficulties) and chronic disease comorbidities, were systematically extracted from patient records. The study utilised routinely collected clinical data, including patient demographics, clinical presentations, documented symptoms, and comorbid conditions. The selection of variables was guided by the availability and consistency of information recorded across facilities, with emphasis on variables routinely and reliably documented in patient records. Data extraction was performed using a structured data collection tool to ensure consistency across facilities.

Diagnosis of dementia was based on the Diagnostic and Statistical Manual of Mental Disorders, Fifth Edition (DSM-5), published by the American Psychiatric Association [17]. The DSM-5 defines dementia (major neurocognitive disorder) as a significant cognitive decline in one or more cognitive domains (for example, memory, language, executive function) that interferes with independence in daily functioning [17]. Diagnoses in this study were made by qualified clinicians using these standardised criteria, ensuring consistency across cases. The symptoms reported in this study were obtained from routine clinical assessments conducted by healthcare practitioners. These assessments included patient self-reports, caregiver reports, and clinician observations documented in medical records.

The Neuropsychiatric Inventory (NPI) framework was used as a guide to categorise behavioural and psychological symptoms extracted from the records, improving consistency in symptom classification [18]. The NPI is a popular tool for both clinical and research purposes, and it helps assess behavioural and psychological symptoms in people with dementia and other neurological disorders [19]. A chronic condition was defined as the presence of at least one of the following conditions: hypertension, diabetes, cardiovascular disease, or chronic kidney disease [15]. Records contained a tick-box for chronic conditions and space for specification; when unspecified, only the presence was noted, but commonly specified conditions are presented in the Results.

### 2.3. Statistical Analysis

Data were analysed using R software, version 4.3.0. Descriptive statistics were presented as frequencies and percentages for categorical variables. Independent variables included age group and gender, while dependent variables comprised dementia-related symptoms and the presence of a chronic disease. Associations between variables were assessed using Pearson’s Chi-square test. A two-sided *p*-value of less than 0.05 was considered statistically significant. No imputation was performed for missing data, as the primary variables of interest were sufficiently complete for analysis.

### 2.4. Ethical Considerations

The study protocol was reviewed and approved by the Walter Sisulu University Faculty of Health Sciences Research Ethics Committee (Approval Number: WSU HREC 102/2025). The requirement for informed consent was waived due to the study’s retrospective nature and the use of anonymised data.

## 3. Results

### 3.1. Participant Characteristics

A total of 260 participants with a confirmed diagnosis of dementia were included in the analysis, the majority being female (59.6%, n = 155). Participants were predominantly older adults, with 64.3% (n = 167) aged ≥71 years, including 30.8% (n = 80) aged 71–80 years and 33.5% (n = 87) aged ≥80 years. Regarding the year of diagnosis, over half of the cases were recorded in the most recent years, with 27.3% (n = 71) diagnosed in 2023 and 27.7% (n = 72) in 2024 (Table 1). A high prevalence of dementia-related symptoms was observed across the study population (Table 2).

### 3.2. Prevalence of Dementia-Related Symptoms and Comorbidities

A high prevalence of dementia-related symptoms was observed across the study population (Table 2). Short-term memory loss and behavioural changes were the most frequent symptoms, affecting 96.9% of participants each. General memory loss was also highly prevalent (91.2%). Communication difficulties were reported in 67.3% of patients, while delusions were present in 57.7%. Chronic disease comorbidity was common, with 75.8% of participants having at least one chronic condition.

### 3.3. Association Between Age Group and Dementia Symptoms

The distribution of dementia-related symptoms across age groups is presented in Table 2. Memory loss was highly prevalent across all age categories, ranging from 87.5% to 95.4%. Similarly, behavioural changes and short-term memory loss were consistently high across all groups (>95%). Although some variation was observed in the prevalence of delusions, communication difficulties and chronic disease across age groups, these differences were not statistically significant. No significant associations were found between age group and any of the assessed symptoms or conditions (all *p*-values > 0.05).

### 3.4. Association Between Gender and Dementia Symptoms

The prevalence of dementia-related symptoms by gender is shown in Table 3. Memory loss affected over 90% of both males (92.4%) and females (90.3%). Behavioural changes and short-term memory loss were similarly prevalent in both groups (≥96%). Communication difficulties were reported in 66.7% of males and 67.7% of females, while delusions were present in 60.0% of males and 56.1% of females. Chronic disease comorbidity was observed in 81.0% of males and 72.3% of females. Despite these differences in proportions, no statistically significant associations were found between gender and any of the assessed symptoms or chronic disease (all *p*-values > 0.05).

## 4. Discussion

This study provides a detailed assessment of dementia symptom burden and comorbidity in a resource-limited South African clinical population. Core cognitive symptoms, particularly short-term memory loss and behavioural changes, were nearly universal, with other manifestations such as general memory loss, communication difficulties, and delusions also highly prevalent. Additionally, three-quarters of participants had at least one chronic disease. Symptom prevalence and comorbidity patterns did not differ significantly across age or gender, highlighting a substantial and consistent clinical burden in these settings and underscoring the need for integrated, person-centred care.

The near-universal cognitive and behavioural symptoms align with core clinical features of dementia and reflect its multifaceted nature, encompassing cognitive, neuropsychiatric, and functional domains [9,10]. These symptoms are not unique to Alzheimer’s disease; they also manifest in vascular, frontotemporal, and other neurocognitive disorders [6]. The high prevalence of delusions and communication difficulties highlights pervasive neuropsychiatric and functional impairments beyond memory deficits, contributing to caregiver burden and reduced quality of life [14,15]. Compared with studies in high-income settings, the prevalence estimates here are at the upper end, likely reflecting the inclusion of patients at moderate to advanced disease stages and the concentration of healthcare access in rural facilities.

Multimorbidity was highly prevalent, with hypertension, diabetes, and cardiovascular disease most commonly specified. Multiple chronic conditions accelerate cognitive decline and complicate management [8,20], particularly in resource-limited settings lacking integrated care pathways. Our findings mirror global and regional studies reporting multimorbidity rates of 65–77% among older adults [21,22]. Although no statistically significant association between comorbidity burden and age was observed, this may reflect early overlap of vascular and metabolic risk factors in dementia pathogenesis. A non-significant trend toward higher comorbidity in males warrants further investigation in larger cohorts. Collectively, these data highlight the importance of holistic patient management addressing both cognitive decline and chronic physical health conditions.

Contrary to some reports suggesting demographic differences in symptomatology [16,23], our study found no significant variation across age or gender, likely reflecting the moderate to advanced disease stage of participants. This uniformity supports universal screening and management rather than age- or gender-specific interventions, facilitating equitable resource allocation and timely care [5,24]. Caregiving strategies, support services, and clinical monitoring should target the full spectrum of symptoms irrespective of demographics.

These results have practical and policy implications. The consistently high symptom burden underscores the need for integrated, interdisciplinary care that combines cognitive, neuropsychiatric, and physical healthcare [25]. Communication support and caregiver guidance are essential [26]. Standardised assessment protocols capturing cognitive, behavioural, and comorbidity data may enable more efficient allocation of scarce resources. For research, longitudinal studies incorporating dementia subtypes, disease severity, and biomarkers are needed to better understand symptom trajectories, interactions with comorbidities, and the effectiveness of integrated care interventions.

### Limitations

Several limitations warrant consideration. The retrospective design relies on routinely documented medical records, which may underreport symptoms that are not systematically assessed. Data on dementia subtype, severity (for example, MMSE or CDR scores), and pharmacologic treatments were unavailable, limiting more granular analysis. Additionally, this single-clinic study may not fully represent other rural or urban populations. Finally, the absence of significant demographic associations may reflect limited statistical power to detect subtle differences. Despite these limitations, the study provides a detailed, real-world snapshot of dementia symptom burden and comorbidity in a resource-limited clinical setting, informing further research and programs.

## 5. Conclusions

Dementia-related symptoms are consistently severe across age and gender in this rural South African clinical population, highlighting the complex intersection of cognitive, neuropsychiatric, and physical health challenges. These findings underscore the urgent need for integrated, person-centred care models addressing both symptom management and multimorbidity. Contemporary evidence shows that traditional care methods focused on specific diseases often fall short for people with multiple chronic conditions [27]. These methods do not consider the complex mix of physical, psychological, and social needs. Instead, person-centred integrated care has come forward as a better approach [27]. It encourages coordinated, holistic, and personalised care across different conditions and services. Recent studies show that the rising problem of multimorbidity raises the risk of fragmented care [28]. This situation calls for integrated models that ensure continuity, coordination, and patient involvement in decision-making for the interventions related to their health. Standardized assessment protocols and interdisciplinary interventions are essential to optimize patient outcomes and support caregivers, particularly in resource-limited settings [29].

Future research should explore dementia subtypes, longitudinal symptom trajectories, and the impact of integrated care strategies, ideally incorporating biomarkers, disease severity measures, and socioeconomic context. Such studies will inform evidence-based clinical management and policy planning, ensuring that interventions are both effective and equitable across diverse populations.

## Figures and Tables

**Table 1:** Participant characteristics (n = 260).

Variable	Frequency (n)	Percentage (%)
Gender		
- Male	105	40.4
- Female	155	59.6
Age group (years)		
- 50–60	30	11.5
- 61–70	63	24.2
- 71–80	80	30.8
- 80+	87	33.5
Year of diagnosis		
- 2019	33	12.7
- 2020	24	9.2
- 2021	39	15.0
- 2022	21	8.1
- 2023	71	27.3
- 2024	72	27.7

**Table 2:** Association between participant age group and dementia symptoms.

Symptom/Condition	Age Group	n (%)	*p*-Value
Memory loss	50–60	28 (93.3)	0.278
	61–70	56 (88.9)	
	71–80	70 (87.5)	
	80+	83 (95.4)	
Change in behaviour	50–60	30 (100.0)	0.484
	61–70	62 (98.4)	
	71–80	76 (95.0)	
	80+	84 (96.6)	
Delusions	50–60	20 (66.7)	0.587
	61–70	37 (58.7)	
	71–80	42 (52.5)	
	80+	51 (58.6)	
Short-term memory loss	50–60	30 (100.0)	0.773
	61–70	61 (96.8)	
	71–80	77 (96.3)	
	80+	84 (96.6)	
Difficulty communication	50–60	22 (73.3)	0.121
	61–70	36 (57.1)	
	71–80	52 (65.0)	
	80+	65 (74.7)	
Chronic disease *	50–60	23 (76.7)	0.876
	61–70	50 (79.4)	
	71–80	59 (73.8)	
	80+	65 (74.7)	

* Commonly specified chronic diseases were hypertension (33%), diabetes mellitus (11%), and dyslipidaemia (43%) among the 197 participants with at least one chronic condition.

**Table 3:** Association between gender and dementia symptoms = 260.

Symptom/Condition	Male (%)	Female (%)	*p*-Value
Memory loss	92.4	90.3	0.566
Change in behaviour	97.1	96.8	0.866
Delusions	60.0	56.1	0.535
Short-term memory loss	97.1	96.8	0.866
Difficulty communication	66.7	67.7	0.856
Chronic disease	81.0	72.3	0.108

## Data Availability

The datasets presented in this article are not publicly available due to privacy and ethical restrictions protecting the confidentiality of study participants. However, anonymised data may be made available to the corresponding author upon reasonable request, subject to approval by the Walter Sisulu University ethics committee. Requests to access the datasets should be directed to M. A. Dlamini: Email: adlamini@wsu.ac.za.

## Abbreviations

The following abbreviations are used in this manuscript:

CDR	Clinical Data Repository
MMSE	Mini-Mental State Examination
